# Eukaryotic Phytoplankton Contributing to a Seasonal Bloom and Carbon Export Revealed by Tracking Sequence Variants in the Western North Pacific

**DOI:** 10.3389/fmicb.2019.02722

**Published:** 2019-11-26

**Authors:** Takuhei Shiozaki, Yuu Hirose, Koji Hamasaki, Ryo Kaneko, Kazuo Ishikawa, Naomi Harada

**Affiliations:** ^1^Earth Surface System Research Center, Japan Agency for Marine-Earth Science and Technology, Yokosuka, Japan; ^2^Department of Applied Chemistry and Life Science, Toyohashi University of Technology, Toyohashi, Japan; ^3^Atmosphere and Ocean Research Institute, The University of Tokyo, Tokyo, Japan; ^4^Arctic Environment Research Center, National Institute of Polar Research, Tachikawa, Japan

**Keywords:** 18S rRNA, eukaryotic phytoplankton, biological pump, coastal diatoms, prasinophytes

## Abstract

Greater diversity of eukaryotic phytoplankton than expected has been revealed recently through molecular techniques, but little is known about their temporal dynamics or fate in the open ocean. Here, we examined size-fractionated eukaryotic phytoplankton communities from the surface to abyssopelagic zone (5,000 m) throughout the year, by tracking sequence variants of the 18S rRNA gene in the western subtropical North Pacific. The oceanographic conditions were divided into two periods, stratification and mixing, between which the surface phytoplankton community differed. During the mixing period, the abundance of large phytoplankton (≥3 μm) increased, with diatoms and putative *Pseudoscourfieldia marina* dominating this fraction. Picophytoplankton (<3 μm) also increased during the mixing period and were dominated by Mamiellophyceae. Taxa belonging to prasinophytes (including *Ps. marina* and Mamiellophyceae) were observed in the epipelagic zone throughout the year, and thus likely seeded the seasonal bloom that occurred during the mixing period. In contrast, diatoms observed during the mixing period mostly represented taxa unique to that period, including coastal species. Numerical particle backtracking experiments indicated that water masses in the surface layer could be transported from coastal areas to the study site. Gene sequences of coastal diatoms were present in the abyssopelagic zone. Therefore, allochthonous species drove the seasonal bloom and could be transported to deep waters. In the abyssopelagic zone, the relative abundance of *Ps. marina* in deep waters was similar to or higher than that of diatoms during the mixing period. Among picophytoplankton, Mamiellophyceae made up a significant fraction in the abyssopelagic zone, suggesting that prasinophytes are also involved in carbon export. Our molecular survey showed that these previously overlooked phytoplankton species could contribute significantly to the seasonal bloom and biological pump in the subtropical open ocean.

## Introduction

Phytoplankton play a key role in shaping marine ecosystem structures and global biogeochemical cycles. Phytoplankton are highly sensitive to environmental changes, which affect their abundance and community structure, and understanding the temporal dynamics of phytoplankton is therefore essential to predicting future global environmental changes (Giovannoni and Vergin, 2012; Winder and Sommer, 2012). Recent molecular techniques have revealed extreme diversity among eukaryotic phytoplankton; most of these are difficult to identify using conventional methods such as microscopic and pigment analyses (Vaulot et al., 2008; Not et al., 2012), while prokaryotic phytoplankton are primarily composed of two genera, *Prochlorococcus* and *Synechococcus*. Comprehensive analysis of diverse eukaryotic phytoplankton has only just begun, and temporal variations in their community structure remain unclear.

We investigated temporal variations in the eukaryotic phytoplankton community using a high throughput sequencing technology, not only in the epipelagic zone but also down to the abyssopelagic zone. Carbon fixed by phytoplankton in oceanic surface waters is exported in part to the ocean’s interior; this process is referred to as the biological pump. The efficiency and strength of the biological pump is controlled by nutrient availability, phytoplankton production, and food web structure (Ducklow et al., 2001; Guidi et al., 2016). The phytoplankton community in deep water is therefore used as an indicator of the surface environment, and of the function of the biological pump (e.g., Alldredge and Gotschalk, 1989; Volkman et al., 1998; Honda and Watanabe, 2010; Harada et al., 2012). Diatoms and coccolithophores are groups of phytoplankton in deep waters that have been well-studied, as they are distributed worldwide; moreover, their mineral shells, made from silicate and carbonate, respectively, are efficiently transferred to deep waters (“ballasting effect”), wherein they are well-preserved (Armstrong et al., 2002; Francois et al., 2002). Other phytoplankton typically lack such mineral shells, which causes difficulty in their morphological identification; thus, less attention has been paid to other taxa.

We examined two different size classes (≥3- or <3-μm) of eukaryotic phytoplankton. Particle size is an important factor determining sinking velocity (Smayda, 1970; Mestre et al., 2018). Diatoms and coccolithophores, which are considered key groups of sinking particles, are generally larger than 3 μm (Paasche, 2002; Tréguer et al., 2018). Thus, the importance of other phytoplankton to the biological pump relative to diatoms and coccolithophores in the same size fraction was assessed. For small phytoplankton, while the sinking rate of individual cells is slow, several mechanisms are known to enhance the sinking rate (Richardson, 2019 and references therein). However, aside from some cyanobacteria, whether some species are preferentially delivered to deep waters remains poorly understood. Here, we track sequence variants (SVs) of the 18S rRNA gene both temporally and vertically. SV analysis, which can resolve single-nucleotide differences, is apparently more suitable for examining spatial and temporal variations within a species compared to operational taxonomic unit (OTU) clusters. To the best of our knowledge, this study is the first characterization of temporal variations of eukaryotic phytoplankton community structure at nearly full depth over the course of a year in the open ocean. Our observations shed new light on the eukaryotic phytoplankton community, in terms of their temporal dynamics and potential significance to the biological pump.

## Materials and Methods

**FIGURE 1:**
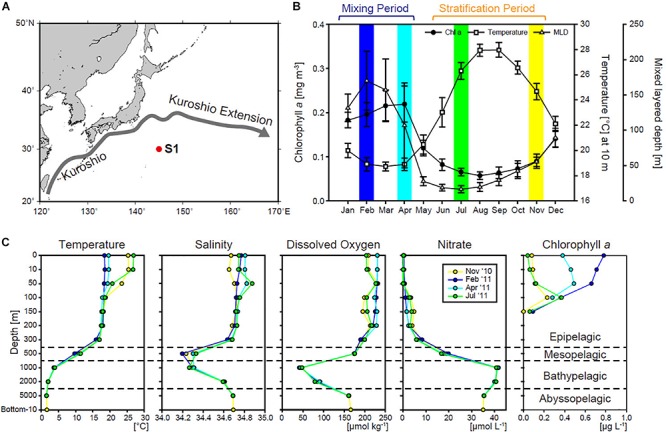
**(A)** Location of station S1 in the northwestern North Pacific Ocean. **(B)** Seasonal variations in satellite-derived (MODIS Aqua) chlorophyll *a* (chl *a*), sea surface temperature, and Argo-derived mixed layer depth (MLD) (Hosoda et al., 2010) at station S1 (1° × 1° area), averaged between January 2003 and December 2012. Colored bars indicate the months in which the cruises took place. **(C)** Vertical profiles of temperature, salinity, dissolved oxygen, nitrate, and chl *a* during each cruise.

Sampling was performed onboard the R/V Mirai at station S1 in the western subtropical North Pacific (30°N, 145°E; Figure 1A) during 4 months in 2010–2011 (November 2010, February 2011, April 2011, and July 2011), corresponding to the cruises MR10-06, MR11-02, MR11-03, and MR11-05, respectively. Station S1 is located in a region of subtropical mode water (Suga and Hanawa, 1990). The mixed layer annually deepens in winter and spring, bringing nutrients to the surface that support seasonal phytoplankton blooms (Figure 1B). Conversely, the shallower mixed layer depth in summer and fall results in surface nutrient depletion and low productivity (Figure 1B). Our four sampling periods corresponded to the mixing (February 2011 and April 2011) and stratification (November 2010 and July 2011) periods, respectively. The results were summarized for comparison of the stratification and mixing periods. We collected samples more intensively above 200 m compared to deeper waters, as the phytoplankton community was expected to change more dramatically in shallower waters; the sampling depths were 0, 5/10, 50, 100, 150, 200, 300, 500, 1,000, 2,000, and 5,000 m. Samples were collected using Niskin-X bottles or a bucket. During the MR10-06 cruise, samples were also collected from 10 m above the bottom (6,074 m). Samples for chlorophyll *a* (chl *a*) were collected only above 200 m. Samples (2–4 L) of DNA from each depth were sequentially size-fractionated using 3-μm polycarbonate filters (Whatman, Maidstone, United Kingdom) followed by filtration through 0.2-μm Millipore Sterivex filter units (EMD Millipore, Darmstadt, Germany). The sample filters were frozen immediately and stored at −80°C until onshore analysis. It should be noted that the small filtration volumes used in this study are a potential source of error with respect to community structure in the large size fraction. Large organisms can be missed when small filtration volumes are used, although organisms in the small fraction were unlikely to be missed due to their expected homogenous distribution.

Temperature, salinity, and dissolved oxygen profiles were measured using an SBE 911 Plus system (Sea-Bird Electronics, Bellevue, WA, United States). The oxygen and salinity sensors were calibrated using bottle data collected during each cruise. Nitrate concentrations were determined colorimetrically on board (Hansen and Koroleff, 1999) using the QuAAtro 2-HR system (SEAL Analytical, Southampton, United Kingdom). Size-fractionated samples of chl *a* were sequentially filtered onto 10-, 3-, 1-, and 0.2-μm polycarbonate filters. Here, we present chl *a* concentrations only for the ≥3- and <3-μm fractions, corresponding to the size-fractionated samples used for DNA analysis. The details of the analytical methods used for environmental variables are provided in the cruise reports^1^.

### DNA Extraction, Sequencing, and Phylogenetic Analysis

Total DNA was extracted using a ChargeSwitch Forensic DNA Purification Kit (Invitrogen, Carlsbad, CA, United States), as described by Kaneko et al. (2016). The V7–V8 region of the 18S rRNA gene was amplified using Tks Gflex DNA Polymerase Low DNA (Takara Bio, Shiga, Japan) with the primers F-1183 (5′-AATTTGACTCAACACGGG-3′) and R-1631 (5′-TACAAAGGGCAGGGACG-3′) (Hadziavdic et al., 2014) attached to the adapters Forward (5′-ACACTCTTTCCCTACA CGACGCTCTTCCGAT-3′) and Reverse (5′-GTGACTGGA GTTCAGACGTGTGCTCTTCCGATCT-3′), respectively. The subsequent procedures are as described by Shiozaki et al. (2018), with minor modifications. Briefly, triplicate PCR products were pooled and purified using the AMPure XP purification kit (Beckman Coulter, Brea, CA, United States), and then indexed with the Nextra XT Index Kit (Illumina, San Diego, CA, United States). The indexed library was purified again using the AMPure XP purification kit and quantified with a Quantus Fluorometer (Promega, Madison, WI, United States). All samples were added to the multiplex pool at equimolar concentrations and then sequenced on the Illumina MiSeq platform (Illumina, San Diego, CA, United States), wherein 300-bp fragments from each end of the libraries were sequenced using the MiSeq Reagent Kit v3 (600 cycles; Illumina, San Diego, CA, United States) with the PhiX control v3 (Illumina, San Diego, CA, United States). Sequenced reads were demultiplexed using MiSeq Reporter v2.6.2 (Illumina, San Diego, CA, United States). The demultiplexed reads were deposited in the DNA Data Bank of Japan Sequence Read Archive under accession number DRA008479.

Community analysis of the reads was performed using the QIIME2 program (ver. 2019.7; Bolyen et al., 2019). Primer sequences were removed using the Cutadapt plug-in (Martin, 2011). The reads were denoised and clustered based on SVs at single-nucleotide resolution using the Deblur plug-in (Amir et al., 2017). The 3′-ends of the forward and reverse sequences were merged and trimmed to 400 bp using the –p-trim-length 400 command. The obtained SVs were searched against the Protist Ribosomal Reference Database (PR^2^ ver. 4.10, Guillou et al., 2013) to discriminate unicellular eukaryotic sequences. Then, the SVs were taxonomically classified with a naïve Bayes classifier trained on reference sequences based on 99% OTUs in SILVA 123 (Quast et al., 2013). We further compared representative sequences to the GenBank database using BLASTn^2^. Samples were subsequently rarefied to the minimum number of reads (6,913 reads) to normalize read counts across samples.

Sequences assigned to phytoplankton were selected for further analysis, and all other sequences were excluded from this study except when calculating the ratio of eukaryotic phytoplankton to total protists (unicellular eukaryotes). The SVs of phytoplanktonic taxa are classified based on the systematics of Not et al. (2012). Specifically, we targeted SVs classified as Archaeplastida, Cryptophyta, Haptophyta, Chlorarachniophyta, and Heterokontophyta. Sequences representing Euglenophyta were not recovered from our samples. The taxa analyzed are known to contain some heterotrophs (Not et al., 2012), and their SVs were excluded from the analysis. The dinoflagellates include a significant fraction of heterotrophic species, as well as many undescribed species (Vaulot et al., 2008; Not et al., 2012). The results of pigment analysis conducted during the same cruises indicated that dinoflagellates accounted for no >4% of the phytoplankton community throughout the year (Fujiki et al., 2016). Therefore, we also excluded dinoflagellates from our analysis.

Non-metric multidimensional scaling (nMDS) with a Bray–Curtis distance matrix and permutational multivariate analysis of variance (PERMANOVA) were used to examine the spatial and temporal variations of the phytoplankton community using the R package vegan (Oksanen et al., 2013). We identified unique SVs for each month, as well as SVs that were common to multiple months in the surface layer and examined the portion of SVs identified during each cruise that was also observed during the following cruise (c.f. Mestre et al., 2018). For this analysis, samples collected at a depth of 5/10 m were used. We examined the extent to which SVs identified in the surface layer were present in deeper zones using the same method. Representative depths for the epipelagic (surface layer), mesopelagic, bathypelagic, and abyssopelagic zones (defined in the section “Results”) were set to 5/10, 500 (or 300 m for the ≥3-μm size fraction in February 2011 and the <3-μm size fraction in April 2011, as the samples collected at 500 m were lost), 1,000, and 5,000 m, respectively.

### Numerical Particle Backtracking Experiment

To trace water masses backward from station S1 to their possible origins, we performed particle backtracking experiments (Ishikawa et al., 2019). Velocity data were derived from the FORA-WNP30 reanalysis product (Usui et al., 2017), which covers the domain of 15–65°N and 117°E–160°W in the North Pacific with a horizontal resolution of 1/10° × 1/10°. The model consists of 54 vertical layers between 0 and 6,300 m depth. Particles were seeded around station S1 (0.25° × 0.25° box) at an interval of 0.025° (441 particles) near our sampling depths (i.e., 0.5, 12, 50, 100, 158, 200, 300, 480, 1,000, 2,000, and 5,000 m). Backtracking was performed at each level using horizontal velocity data and the movement of all particles at all depths was calculated for the 90 days prior to the sampling date for each cruise. The random walk process was not incorporated into these experiments for simplicity.

## Results

### Environmental Variables

Cluster analysis of environmental data demonstrated that water masses differ by depth (Supplementary Figure S1). We defined the depths of ≤300, 500, 1,000–2,000, and 5,000–6,074 m as the epipelagic, mesopelagic, bathypelagic, and abyssopelagic zone, respectively. Note that 2,000 m was rather close to 5,000 m in the cluster analysis, probably due to the similarity of the environmental parameters used at the two depths (Figure 1C), but here we follow the standard oceanographic definition of the bathypelagic zone as depths between 1,000 and 4,000 m). In the epipelagic zone, the upper 50 m (upper 100 m in February 2011) clustered separately from the waters below, and was defined as the surface layer.

Temperature in the surface layer changed markedly between the stratification and mixing periods (Figure 1C). Surface temperature increased to 25.3 and 26.9°C during the stratification period (November 2010 and July 2011, respectively). Temperature was constant (∼20°C) vertically from the surface to 300 m during the mixing period (February 2011 and April 2011). Salinity, dissolved oxygen, and nitrate also showed temporal variations in the surface layer, but did not clearly differ among months below the mesopelagic zone; temperature was also constant at those depths. During the mixing period, the nitrate concentration in the surface layer increased to 1.01 μmol L^–1^ in February 2011, but the maximum value observed was only 0.14 μmol L^–1^ in April 2011. Nitrate varied from 0.03 to 0.19 μmol L^–1^ during the stratification period. Deeper than the mesopelagic zone, a salinity minimum was observed at 500 or 1,000 m, which is a feature of North Pacific intermediate water (Talley, 1993). The oxygen minimum zone was located at 1,000 m, within the bathypelagic zone. The nitrate concentration was highest in the oxygen minimum zone and decreased with depth. The surface chl *a* concentration increased during the mixing period, presumably due to the seasonal bloom in our study region. The chl *a* concentration showed a subsurface maximum except in February 2011, when its maximum value was 0.78 μg L^–1^ at the surface. Chl *a* of the ≥3 μm fraction increased markedly in February 2011, accounting for about half of the total chl *a* (Supplementary Figure S2). The abundance of large (≥3 μm) phytoplankton in the surface layer was higher in April 2011 than in November 2010 or July 2011. In July 2011, large phytoplankton increased at the depth of the subsurface chl *a* maximum (SCM). Sediment trap experiments performed during the same cruises showed that the organic carbon flux increased during the mixing period compared to the stratification period (Honda et al., 2016), suggesting the efficient transport of phytoplankton from surface layer to deep waters during the mixing period.

### Particle-Backtracking Experiments

**FIGURE 2:**
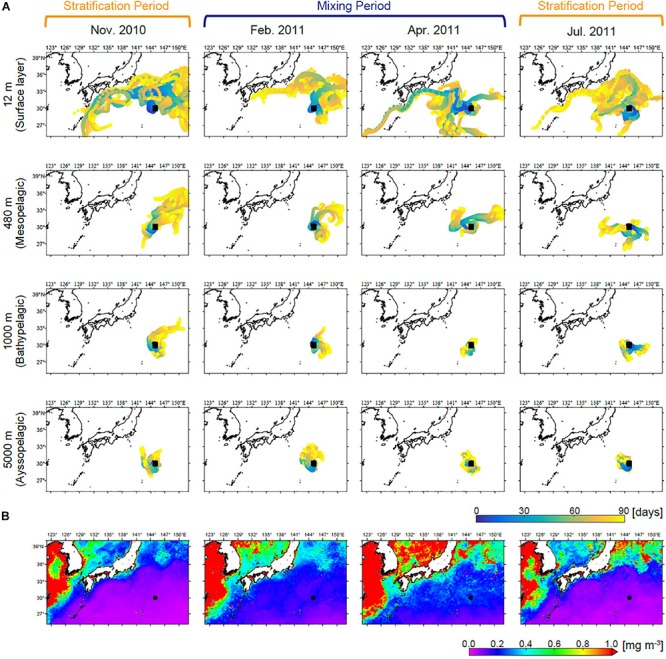
**(A)** Back trajectories of particles present at station S1 at depths of the 12 (surface layer), 480 (mesopelagic zone), 1,000 (bathypelagic zone), and 5,000 m (abyssopelagic zone) over the 90 days prior to sampling each month. Back trajectories at the other depths are shown in Supplementary Figure S3. **(B)** Satellite-derived (MODIS Aqua) chl *a* averaged over the 90 days prior to sampling each month.

Backtracked particle trajectories from station S1 showed similar temporal patterns in each zone (Figure 2A and Supplementary Figure S3). Some water masses in the epipelagic zone at station S1 originate in areas along the coast of Japan, from which they may be entrained into the Kuroshio and transported to the sampling site. On the other hand, below the mesopelagic zone, water movements were relatively small and the origins of water masses were generally estimated to be near the station.

### Sequencing Analysis

**FIGURE 3:**
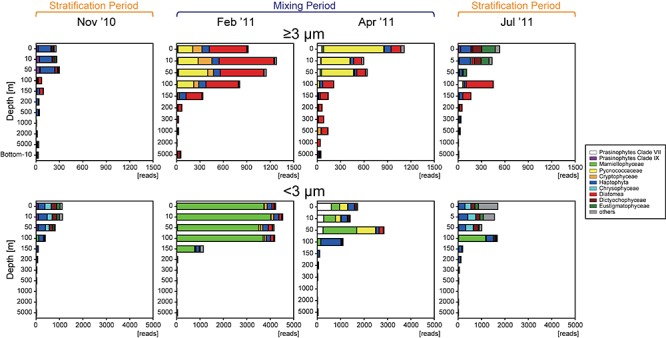
Vertical distributions of the total number of eukaryotic phytoplankton sequences in each fraction during each season. Major groups are shown in color and listed in the legend.

**FIGURE 4:**
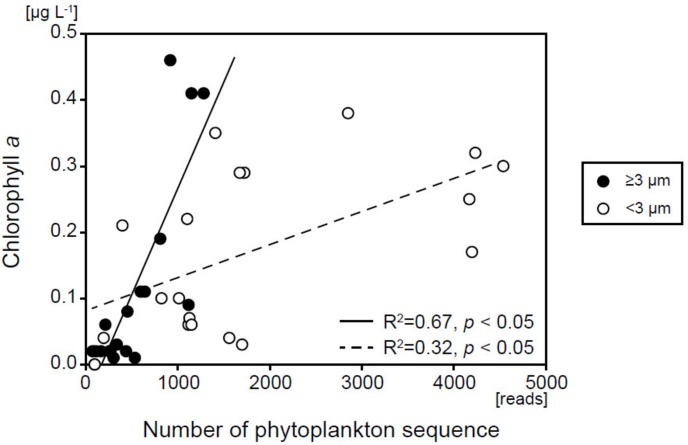
Relationship between the number of eukaryotic phytoplankton sequences and chl *a* concentration for each size fraction.

Sequences belonging to phytoplankton were recovered throughout the year at all depths, from the surface layer to the abyssopelagic zone, and in both size fractions. The number of SVs in the ≥3-μm size fraction (33–93) was comparable with that in the <3-μm size fraction (56–132) in the surface layer (Supplementary Figure S4). In contrast, the numbers of sequence reads in the surface layer were always higher in the <3-μm size fraction than in the ≥3-μm size fraction (*t*-test, *p* < 0.05) (Figure 3), indicating that the ratio of phytoplankton abundance to total protists was higher in the <3-μm size fraction (11.9–65.6%) than in the ≥3-μm size fraction (4.15–20.7%). The number of SVs and sequence reads in both size fractions was greatest in the surface layer and decreased with depth in all months. Sequence reads in both size fractions were significantly and positively correlated with the corresponding fraction of chl *a* (*p* < 0.05) (Figure 4), indicating that the total read number roughly represented the abundance of phytoplankton.

**FIGURE 5:**
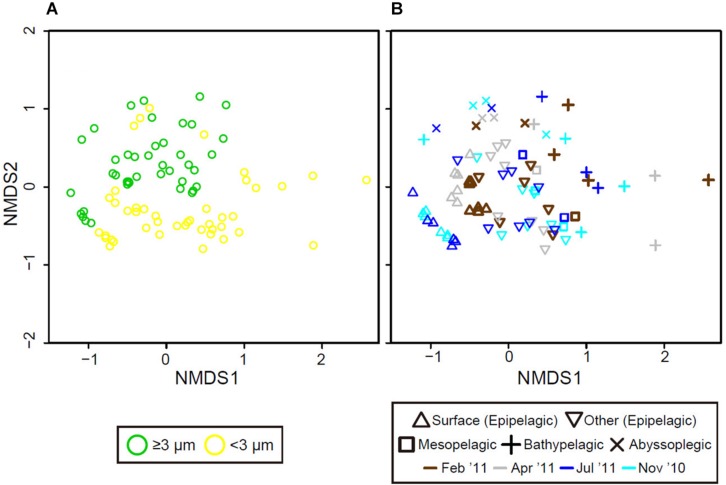
Grouping of eukaryotic phytoplankton communities based on non-metric multidimensional scaling (nMDS) according to community similarity (Bray–Curtis distance), differentiated by **(A)** size fraction (color), **(B)** water depth (shape), and month (color).

Non-metric multidimensional scaling analysis showed that the samples clustered by size fraction (PERMANOVA, *p* < 0.0001), water column (*p* < 0.0001), and two periods (*p* < 0.0001), with a stress value of 0.200 (Figures 5A,B). Eukaryotic phytoplankton communities in the ≥3-μm size fraction were significantly separated from those in the <3-μm size fraction. Furthermore, the phytoplankton communities in the epipelagic zone formed a separate cluster from those in the bathypelagic and abyssopelagic zones, while communities near the bottom of the epipelagic zone were similar to those in the mesopelagic zone. Temporal differences in eukaryotic phytoplankton communities between the mixed and stratified periods were especially prominent in the surface layer.

**FIGURE 6:**
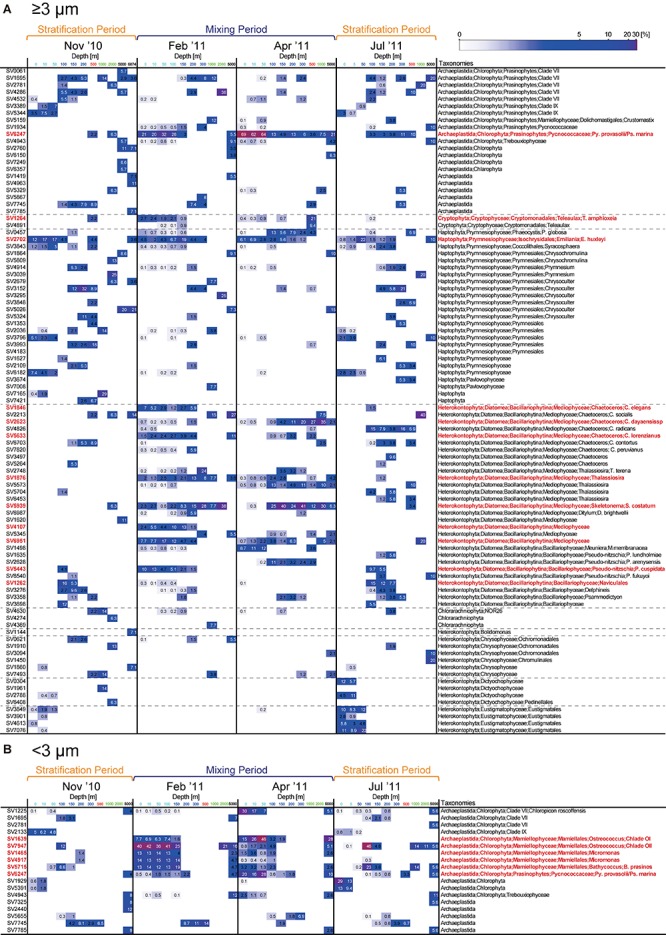
Heatmap of the relative abundance of representative sequence variants (SVs; ≥5% of total reads at a given depth) in the **(A)** ≥3-μm and **(B)** <3-μm size fractions. For the <3-μm size fraction, only SVs assigned to Archaeplastida are shown, while other SVs are shown in Supplementary Figure S5. Relative abundance is shown in white when it is >5%. The SVs noted in the text are shown in red.

Representative SVs with sequence reads accounting for ≥5% of total reads in the ≥3- or <3-μm size fraction in the epipelagic zone belonged to numerous groups (Figures 3, 6, and Supplementary Figure S5). Among Archaeplastida, the SVs were mainly assigned to clade VII (Guillou et al., 2004) and IX (Shi et al., 2009) of the prasinophytes, Pycnococcaceae and Mamiellophyceae. Most representative SVs within Haptophyta were assigned to Prymnesiophyceae. Mediophyceae (Diatomea), Bacillariophyceae (Diatomea), Dictyochophyceae, Eustigmatophyceae, and Chrysophyceae were dominant among the representative SVs of Heterokontophyta. There were few single SVs that made up ≥5% of the total reads for Cryptophyta and Chlorarachniophyta in the epipelagic zone. Sequences assigned to Diatomea were found mainly in the ≥3-μm size fraction, and rarely in the <3-μm size fraction. On the other hand, sequences of Mamiellophyceae were found mainly in the <3-μm size fraction. The other groups named above were observed in both size fractions.

### Temporal Variations of the Eukaryotic Phytoplankton Community in the Epipelagic Zone

**FIGURE 7:**
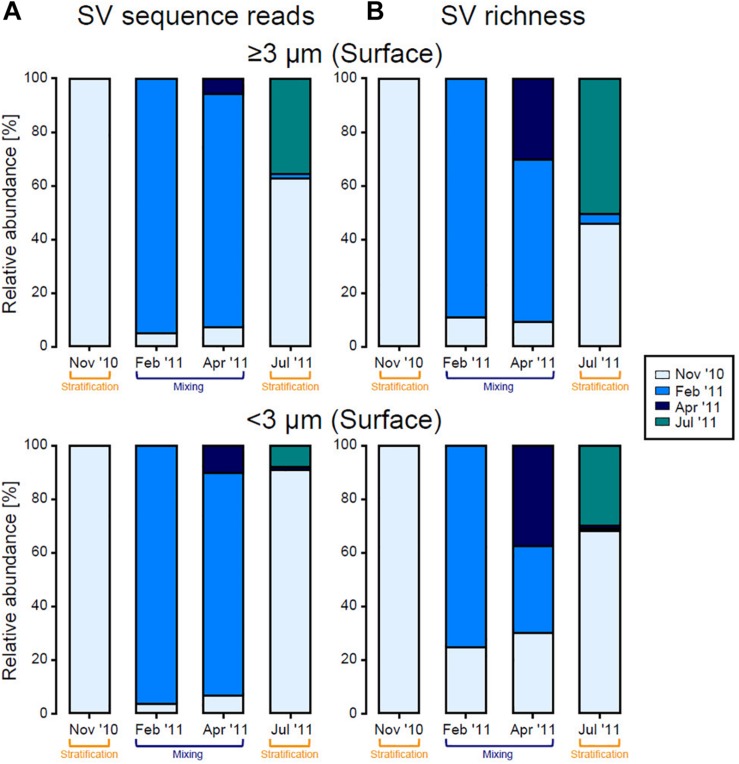
Contribution of eukaryotic phytoplankton sequence reads originating in each month, for each size fraction in the surface layer, calculated on the basis of **(A)** SV sequence reads and **(B)** SV richness based on the number of sequence reads and presence/absence (binary) data, respectively. SVs observed in November 2010 were set to 100%, shown by the first bar (light blue), and ratios of that value to total reads in each subsequent months are shown by the other bars. SVs that were first detected in February 2011, April 2011, and July 2011 are indicated by blue, dark blue, and green bars, respectively.

Haptophyta were observed throughout the year, and the major SV within Haptophyta (SV2702) did not change during the study period. In contrast, most other groups differed in relative abundance among months. Thus, a clear difference in eukaryotic phytoplankton community structure was observed between the stratification (November 2010 and July 2011) and mixing (February 2011 and April 2011) periods, especially in the surface layer in both size fractions. Based on SV sequence reads, more than half of the SVs recorded in July 2011 were present in the surface phytoplankton community in November 2010 (Figure 7A). During the mixing period, most SVs observed in April 2011 were also present in February 2011. At the same time, our results also showed that new taxa continuously joined the community over time, and this trend was clearer when calculated on the basis of SV richness (Figure 7B).

### Stratification Period (November 2010 and July 2011)

The vertical structure of the phytoplankton community in November 2010 was similar to that in July (Figure 3). In the surface layer, sequence reads representing Dictyochophyceae, Eustigmatophyceae, Chrysophyceae, and clade XI prasinophytes increased during the stratification period compared to the mixing period. The abundance of Diatomea relative to total sequence reads increased below 100 m in the ≥3-μm size fraction, reaching 54.8 and 76.1% in November 2010 and July 2011, respectively, and the dominant group within Diatomea was Bacillariophyceae (Figure 6).

### Mixing Period (February 2011 and April 2011)

During the mixing period, Diatomea, Cryptophyceae, Pycnococcaceae, Mamiellophyceae, and clade VII prasinophytes were relatively abundant, and these taxa were rarely observed in the surface layer during the stratification period (Figure 3). Although the ratios of commonly observed SVs to the total community were similar between February 2011 and April 2011 (Figure 7), the dominant taxa in both size fractions differed between these 2 months in the surface layer. Sequence reads representing Diatomea, Cryptophyceae, Pycnococcaceae, and Haptophyta increased in the ≥3-μm size fraction in February 2011, among which Diatomea was the most dominant taxon (48.4–54.8% in total). In the <3-μm size fraction in February 2011, Mamiellophyceae was the dominant taxon (83.4–88.5%). Meanwhile, the dominant SVs in the ≥3-μm size fraction of the surface layer during April 2011 belonged to Pycnococcaceae (61.5–69.0%). Sequence reads for Mamiellophyceae in the <3-μm size fraction decreased in April 2011, and those for Pycnococcaceae and clade VII prasinophytes increased.

Heatmaps were constructed to show the detailed temporal patterns of phytoplankton dynamics in the epipelagic zone (Figure 6 and Supplementary Figure S5). Most representative SVs of Diatomea identified from the surface layer in February 2011 belonged to Mediophyceae, which includes the genera *Chaetoceros*, *Thalassiosira*, and *Skeletonema*, while SV5443 was assigned to the genus *Pseudo-nitzschia* in Bacillariophyceae. Among SVs belonging to Diatomea that were relatively abundant in the surface layer during the mixing period, the sequences SV1846, SV1876, SV4107, SV5633, SV5939, and SV6951 were not recovered from the epipelagic zone on the latest cruise in the year, i.e., during the stratification period (November 2010). SV1846, SV5633, and SV5939 were identical to the 18S rRNA genes of *Chaetoceros elegans*, *Chaetoceros lorenzianus*, and *Skeletonema costatum*, respectively, which are known to be coastal diatoms. The major taxa within Pycnococcaceae and Mamiellophyceae observed during the mixing period were also detected in November 2010. Prasinophytes are one of the key taxonomic groups during the mixing period. In the surface layer, SV6247 which was identical to 18S rRNA genes of *Pycnococcus provasolii* and *Pseudoscourfieldia marina* accounted for 20–32 and 62–69% in total sequence reads of large phytoplankton in February 2011 and April 2011, respectively. Within the small fraction, the relative abundance of SV6247 increased by 16–28% in April 2011. Among Mamiellophyceae, *Ostreococcus* (SV7947), *Micromonas* (SV1465 and SV4917), and *Bathycoccus* (SV5715) were the dominant taxa in the surface picophytoplankton community in February 2011. The major SV assigned to *Ostreococcus* shifted from SV7947 to SV1639 from February 2011 to April 2011, which were identical to 18S rRNA genes of *Ostreococcus* clade OII and clade OI (Demir-Hilton et al., 2011), respectively.

### Connectivity of Phytoplankton Communities in the Epipelagic Zone to Deeper Waters

**FIGURE 8:**
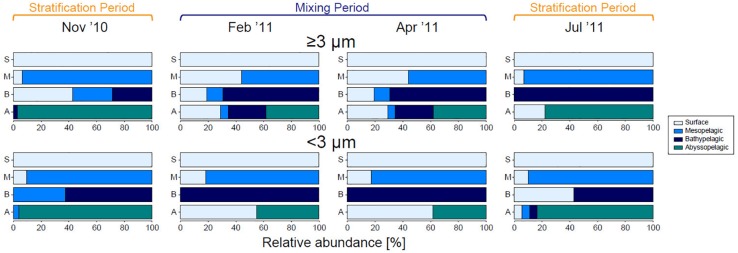
Contribution of eukaryotic phytoplankton sequence reads originating in each layer/zone, for each size fraction during each month. SVs observed in the surface layer were set to 100%, shown by the first bar (light blue), and ratios of that value to total reads in deeper zones are shown below. SVs that were first detected in the mesopelagic, bathypelagic, and abyssopelagic zones are indicated by blue, dark blue, and green bars, respectively.

The contribution of phytoplankton communities in the surface layer to those in deeper waters clearly differed between the stratification and mixing periods in both size fractions (Figure 8). Most sequence reads of SVs originating from the surface layer were relatively abundant at all depths in the large fraction during the mixing period. Meanwhile, during the stratification period, SVs in the large fraction from the surface layer were not observed in the abyssopelagic zone in November 2010, and were rarely observed from the mesopelagic to bathypelagic zones in July 2011. In the small fraction, SVs in the surface layer did not found in the bathypelagic zones, but their relative abundance increased in the abyssopelagic zone during the mixing period; this pattern was not observed during the stratification period. The contribution of the phytoplankton community in the surface layer to those in deeper waters showed a similar pattern in the analysis based on SV richness (data not shown) to that based on sequence reads.

The vertical distribution of representative SVs in the large fraction can be categorized into the following three patterns: (1) occurrence from the epipelagic zone to the bathypelagic or abyssopelagic zone, (2) occurrence only in the epipelagic zone, and (3) occurrence only below the mesopelagic zone. SV6247 (*Py. provasolii*/*Ps. marina*), SV2702 (*Emiliania huxleyi*), and SVs belonging to Mediophyceae of Diatomea (SV1876, SV2623, SV5633, SV5939, and SV6951) occurred sequentially from the surface layer to the bathypelagic or abyssopelagic zone. On the other hand, SVs in the small fraction were rarely sequentially distributed from the surface layer to deeper zones, with the exception of SV3293 and SV6951. Interestingly, some dominant groups of SVs in the small fraction from the surface layer during the mixing period (SV1465, SV1639, SV4917, SV5715, and SV7947), which belonged to Mamiellophyceae, were not present in the mesopelagic zone but appeared in the bathypelagic and abyssopelagic zones. SVs that occurred only below the mesopelagic zone represented the groups Archaeplastida, Haptophyta, *Bolidomonas*, Chlorarachniophyta, Chrysophyceae, Diatomea, and Dictyochophyceae.

## Discussion

### Temporal Variations in the Eukaryotic Phytoplankton Community of the Epipelagic Zone

Temporal variations in the phytoplankton community in bulk water were determined through pigment analysis during the same cruises, but from different depths within the epipelagic zone (Fujiki et al., 2016). Pigment analysis showed that the relative abundance of diatoms within the total phytoplankton community increased during the mixing period (especially in February 2011) compared to the stratification period, and prasinophytes exhibited a similar seasonal pattern. In contrast, haptophytes and chrysophytes occurred continuously throughout the year. The seasonal variations in the eukaryotic phytoplankton community determined through pigment analysis were similar to those identified using 18S rRNA gene sequence analysis. Although cryptophytes were not targeted in pigment analysis (Fujiki et al., 2016), they are known to be abundant in mesotrophic regions but not in oligotrophic waters (Fuller et al., 2006; Vaulot et al., 2008). Similarly, the sequence reads of SVs belonging to Cryptophyta increased in the nutrient-rich surface water of February 2011.

Molecular information allows higher-resolution analysis of phytoplankton communities compared to traditional methods. Treush et al. (2012) examined seasonal variations in the eukaryotic phytoplankton community by applying molecular techniques at the Bermuda Atlantic Time-series Study (BATS) station, which is located in the western North Atlantic subtropical gyre. The eukaryotic phytoplankton community at BATS changed markedly between the mixing and stratification periods, and therefore has similar characteristics to station S1 in this study. Their qPCR analysis targeted three taxa belonging to Mamiellales, i.e., *Ostreococcus*, *Micromonas*, and *Bathycoccus*, and demonstrated that *Ostreococcus* was the most dominant representative of the Mamiellales, followed by *Micromonas* and *Bathycoccus*, which was consistent with our sequencing results for the mixing period. *Ostreococcus* contains two major clades (OI and OII) in the marine environment (Demir-Hilton et al., 2011). OI and OII are referred to as the coastal and oceanic clades, respectively, and have different geographical distributions (Demir-Hilton et al., 2011; Clayton et al., 2017). Although the two clades co-occurred during the mixing period, the number of sequence reads of clade OII decreased sharply from February 2011 to April 2011. Clades OI and OII in the Kuroshio extension region reportedly alter their population structure due to meso- and fine-scale physical dynamics (Clayton et al., 2017). The particle backtracking experiments showed that water masses in the surface layer could be delivered from the north in February 2011, while they arrived mostly from the west in April 2011. The differing temporal patterns of the two clades thus might be attributable to inflow of water masses from different directions to station S1.

The eukaryotic phytoplankton community in the surface layer changed considerably over time from the stratification period (November 2010) to the mixing period (February 2011) (Figures 6, 7). Treush et al. (2012) reported a similar periodicity based on terminal restriction fragment length polymorphism (T-RFLP) analysis and suggested that seed populations present in the SCM during the stratification period could trigger surface blooms during the subsequent mixing period. Indeed, *Ostreococcus* (especially clade OII), *Micromonas*, and *Bathycoccus*, which were major eukaryotic phytoplankton during the mixing period, occurred in the SCM during the stratification period, and thus likely seeded blooms. Our sequencing data showed that diatom blooms did not always follow this pattern, although SV1262 (*Naviculales*) and SV5443 (*Pseudo-nitzschia cuspidata*) did. Most SVs assigned to Diatomea first emerged during the mixing period. Although we could not exclude the possibility that our sequencing depth was insufficient for detection of seed populations in the SCM during the stratification period, the bloom could also have been initiated by seeds delivered from another area. Our results showed that sequences SV1846, SV5633, and SV5939, which emerged in the surface layer and were relatively abundant during the mixing period, were identical to the 18S rRNA genes of *C. elegans*, *S. costatum*, and *C. lorenzianus*, respectively, which are known to be coastal diatom species. Additionally, sequence SV1264 was identical to the 18S rRNA gene of *Teleaulax amphioxeia*, which is a coastal member of the Haptophyta. These results suggest a contribution of coastal species to the seasonal algal bloom in the open ocean. The presence of coastal diatoms in the oceanic region was also reported based on a microscopic study in the western North Pacific (Sugie and Suzuki, 2017). The surface water masses observed at station S1 were simulated to flow from near the coast of Japan, allowing for the delivery of coastal species. Our simulation showed the same trend of surface water movement throughout the year. This result can be interpreted as showing that coastal species cannot grow in the nutrient-poor environment of the study site during stratification, although they may be delivered to the oceanic region during all seasons. This possibility was suggested by the distribution of low surface chl *a* during the stratification period (Figure 2B).

During the time from the mixing period (April 2011) to the subsequent stratification period (July 2011), surface eukaryotic phytoplankton communities in both size fractions again changed dramatically, becoming similar to communities observed during the previous stratification period (November 2010) (Figure 7). Interestingly, many SVs occurred in the epipelagic zone throughout the year, suggesting that those species likely survived through the mixing period and flourished again during the stratification period (Figure 6). Alternatively, as the numerical simulation indicated, recruitment from outside the study region may have occurred. SVs that first appeared in July 2011 were therefore considered allochthonous species.

### Eukaryotic Phytoplankton That May Contribute to the Biological Pump in the Subtropical Region

Numerical particle backtracking experiments demonstrated that water movement was much slower below the mesopelagic zone than in the epipelagic zone. The spatial range of lateral advection below the mesopelagic zone was within a region of consistent surface chl *a* concentration. The surface chl *a* concentration can be considered an index of phytoplankton community structure (Hirata et al., 2011). Therefore, even if our samples collected from deep waters were influenced by lateral advection, the sinking particles we caught are assumed to have derived from an epipelagic zone that is similar to that at station S1. One limitation of our approach is that it cannot detect particles without DNA, such as diatom frustules. Therefore, our results might not reflect the entire eukaryotic phytoplankton community below the mesopelagic zone.

The present study suggests that large eukaryotic phytoplankton can be efficiently transported to the abyssopelagic zone, especially during the mixing period (Figure 8). Coccolithophores and diatoms are known to contribute strongly to the biological pump (Armstrong et al., 2002; Francois et al., 2002). SVs assigned to *E. huxleyi* (SV2702) and members of the Diatomea (SV1876, SV2623, SV5633, SV5939, and SV6951) were relatively abundant from the surface layer to the bathypelagic or abyssopelagic zone, indicating that these species were exported to deeper waters when their abundance increased in the surface layer. Among diatoms, the taxa represented in sequences from deep waters were confined to the group Mediophyceae. The diatom species *C. dayaensis*, *C. lorenzianus*, and *S. costatum*, whose 18S rRNA genes were identical to SV2623, SV5633, and SV5939, respectively, generally occur in coastal environments, and can enter a resting stage. Some coastal diatoms have life cycle strategies that involve resting stage cells. These cells are formed under unfavorable environment conditions, sink to the sediment, and then germinate, causing blooms when conditions are favorable after resuspension through vertical mixing (McQuoid and Hobson, 1996). The resting stage cells sink faster than active cells. Although our approach cannot determine whether the detected SV represents a resting stage cell or not, their presence in deep waters might be related to their life cycle. The sinking rate of diatoms is related to the life strategy of aggregate formation, as well as to their cell size and the Si/C ratio of their cells, which can vary depending on the macro- and micronutrient levels in the environment (Tréguer et al., 2018 and references therein). In our DNA analysis, the diatom class Bacillariophyceae was rarely observed below the mesopelagic zone, which might be related to those factors. However, their frustules may sink to deep waters without DNA. For *E. huxleyi*, the nutrient environment does not substantially affect the cells’ sinking rate (Muggli et al., 1996).

SV6247, which was identical to the 18S rRNA gene of *Py. provasolii/Ps. marina*, became dominant in the surface layer during the mixing period and was subsequently found down to the abyssopelagic zone. The 18S rRNA gene sequence of *Py. provasolii* is known to be almost the same as that of *Ps. marina* (containing one substitution and two gaps among 1,760 nucleotides), and thus Fawley et al. (1999) proposed that they should be placed in the same family despite their cell size differing considerably. The cell size of *Py. provasolii* is generally <3-μm (Guillard et al., 1991), whereas that of *Ps. marina* is over 10 μm (Moestrup and Throndsen, 1988). SV6247 was mainly recovered in the ≥3-μm size fraction in February 2011, and was present in both size fractions in April 2011. These results suggested that *Ps. marina* was present throughout the mixing period, while *Py. provasolii* was rare in February 2011 and became a major constituent of the small phytoplankton in April 2011. Most previous studies on eukaryotic phytoplankton based on molecular approaches have focused on pico-sized (<3-μm cell size) species (Shi et al., 2009; Rii et al., 2016; Kataoka et al., 2017; Wu et al., 2017), and did not observe sequences assigned to *Py. provasolii*, indicating that *Py. provasolii* is probably a minor species in the oceanic environment. Meanwhile, previous studies rarely focused on larger size fractions (≥3 μm), and thus might have overlooked the occurrence of *Ps. marina*. In the large fraction, the relative abundance of SV6247 from the surface layer to the abyssopelagic zone was similar to or higher than that of *E. huxleyi* and diatoms, which suggests a prominent role of *Ps. marina* in the biological pump. The ecology of *Ps. marina* remains poorly understood. Considering its importance, the ecology of this species should be further examined in future research.

Sequence variants detected in the small fraction from the surface layer were also recovered from the bathypelagic and/or abyssopelagic zones, indicating that eukaryotic picophytoplankton contributed significantly to the biological pump. This trend was prominent especially when the surface chl *a* concentration increased during the mixing period. Picophytoplankton are known to reach sinking velocities as high as those of large phytoplankton via several mechanisms (Waite et al., 2000; Richardson and Jackson, 2007; Richardson, 2019). Richardson and Jackson (2007) showed that the contribution of picophytoplankton to the biological pump was proportional to their total net primary production, consistent with our finding. However, while the dominant SVs in the large fraction from the surface were continuously observed from the surface to the bathypelagic and/or abyssopelagic zones, the vertical distribution of SVs in the small fraction was discontinuous. This discrepancy might occur because picophytoplankton are not individually exported from the surface, but instead are rapidly transported to deep waters as aggregates, or with carriers such as zooplankton or settling detritus, which are difficult to detect through water sampling (Riley et al., 2012; Richardson, 2019).

### Eukaryotic Phytoplankton That Do Not Occur in the Epipelagic Zone

Some SVs were not found in the epipelagic zone, but were observed below the mesopelagic zone throughout the year (although they were identified as phytoplankton). This result may be due to (1) the SVs traveling with deep water circulation or (2) the SVs exhibiting a mixotrophic or heterotrophic strategy. Water masses below the mesopelagic zone were relatively stable over the study period. However, we cannot not ignore the possibility that slowly decomposing phytoplankton transported to our study site. Chrysophyta and Haptophyta are known to include species with mixotrophic and heterotrophic strategies (Thomsen et al., 1994; Not et al., 2012). Although we excluded known heterotrophs from our analyses, our dataset could still contain unknown heterotrophs. Pernice et al. (2016) reported that Chrysophytes were major members of bathypelagic microbial communities worldwide, and thrived as bacteriovores.

## Conclusion

The present study documents drastic temporal changes in the surface eukaryotic phytoplankton community in the subtropical region of the northwestern North Pacific Ocean. Our 18S rRNA gene sequencing analysis and numerical simulation revealed that these temporal changes involved not only the indigenous phytoplankton community, but also phytoplankton delivered from other regions. The allochthonous phytoplankton included many taxa of coastal origin, which contribute significantly to the biological pump in this subtropical ocean. Although transport of coastal organisms to oceanic regions by eddies and filaments has been reported (Lin et al., 2010; Stramma et al., 2013), little is known about their contribution to seasonal blooms in the open ocean. Major theories of algal blooms in the open ocean implicitly assume that blooms are seeded by autochthonous phytoplankton (Behrenfeld and Boss, 2014). Our study was conducted over only 1 year; further studies are needed to examine the replicability of our findings.

Furthermore, our results distinguish some taxa important roles in the biological pump of our study region, including organisms outside of the typical taxa known to utilize the ballasting effect, such as diatoms and coccolithophores (Armstrong et al., 2002; Francois et al., 2002). We found that prasinophytes, including putative *Ps. marina*, *Ostreococcus*, *Micromonas*, and *Bathycoccus*, sank to the abyssopelagic zone when they were dominant in the surface layer. Prasinophytes are distributed extensively from subtropical to polar regions (Guillou et al., 2004; Monier et al., 2016), and hence may contribute significantly to the biological pump worldwide.

## Data Availability Statement

The datasets generated for this study can be found in the DNA Data Bank of Japan Sequence Read Archive number DRA008479.

## Author Contributions

TS, KH, and NH designed the experiment. RK and KH collected the samples at sea. TS and YH analyzed the DNA data. KI performed the particle-backtracking experiment. TS prepared the manuscript with contributions from all co-authors. All authors approved the final submitted manuscript.

## Conflict of Interest

The authors declare that the research was conducted in the absence of any commercial or financial relationships that could be construed as a potential conflict of interest.

## Footnotes

1 http://www.godac.jamstec.go.jp/catalog/doc_catalog/e/

2 https://blast.ncbi.nlm.nih.gov
