# Supplementary material for: Eukaryotic Phytoplankton Contributing to a Seasonal Bloom and Carbon Export Revealed by Tracking Sequence Variants in the Western North Pacific
Source: Front Microbiol. 2019 Nov 26;10:2722. doi: 10.3389/fmicb.2019.02722 (PMC6902033; doi:10.3389/fmicb.2019.02722)
Supplement: Supplementary file 1 [file Image_1.pdf]

Supplementary Material for

Eukaryotic phytoplankton contributing to a seasonal bloom and carbon export revealed by tracking sequence variants in the western North Pacific

Takuhei Shiozaki[1], Yuu Hirose[2], Koji Hamasaki[3], Ryo Kaneko[4], Kazuo Ishikawa[3], Naomi Harada[1]

[1] Earth Surface System Research Center, Japan Agency for Marine-Earth Science and Technology, Yokosuka, Japan

[2] Department of Applied Chemistry and Life Science, Toyohashi University of Technology, Aichi, Japan

[3] Atmosphere and Ocean Research Institute, The University of Tokyo, Chiba, Japan

[4] Arctic Environment Research Center, National Institute of Polar Research, Tachikawa, Tokyo, Japan

Contents of this file

Figures S1, S2, S3, S4, and S5

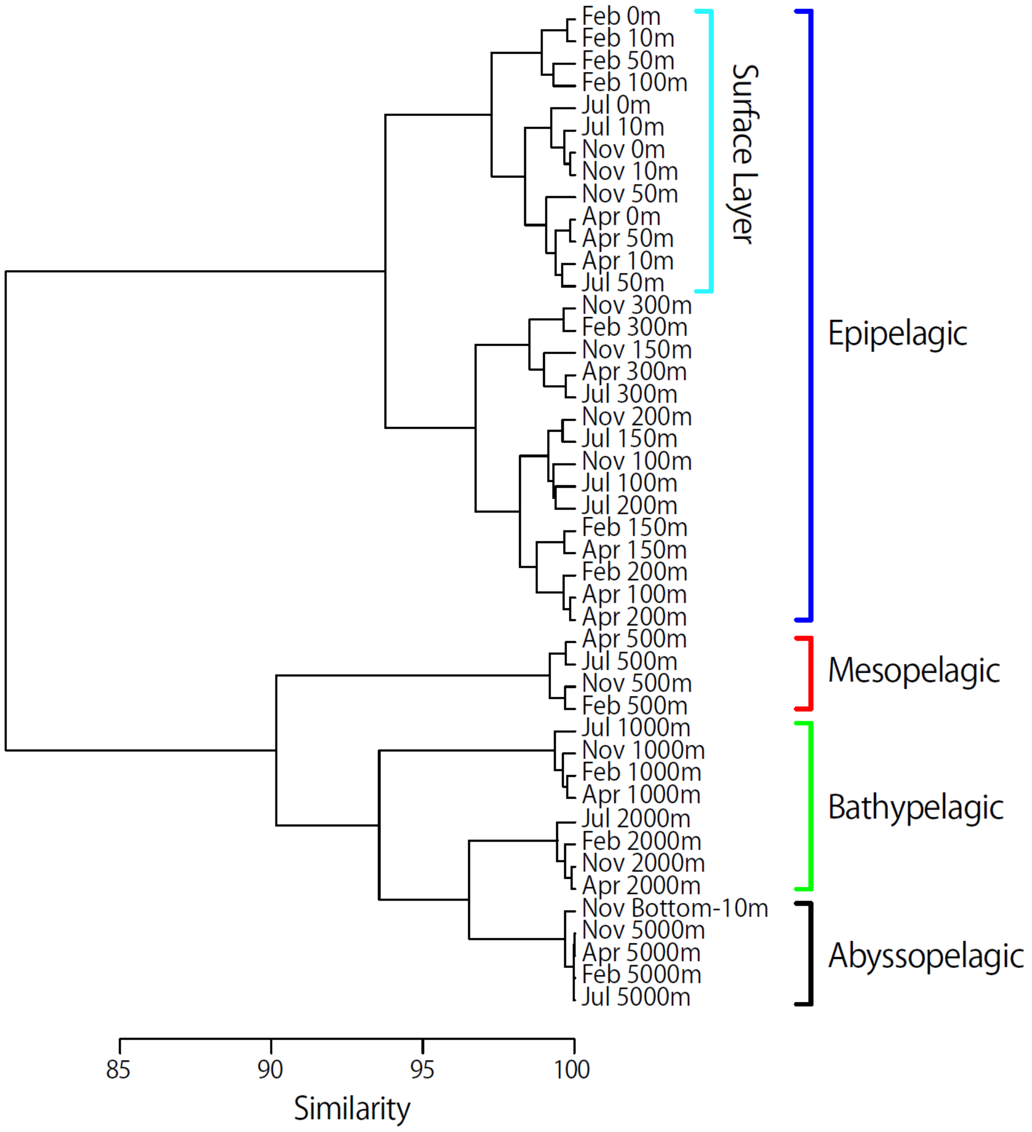

Fig S1. Hierarchical clustering based on the Bray-Curtis similarity of samples used for DNA analysis with environmental variables (temperature, salinity, dissolved oxygen, and nitrate concentration).

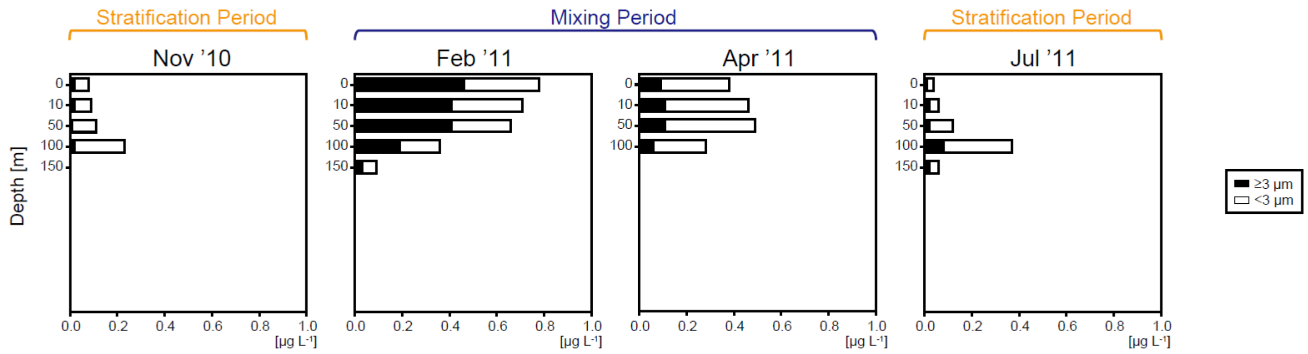

Fig S2. Vertical distribution of the chl *a* concentration in each month. Closed and open bars indicate the ≥ 3-µm and < 3-µm fractions, respectively.

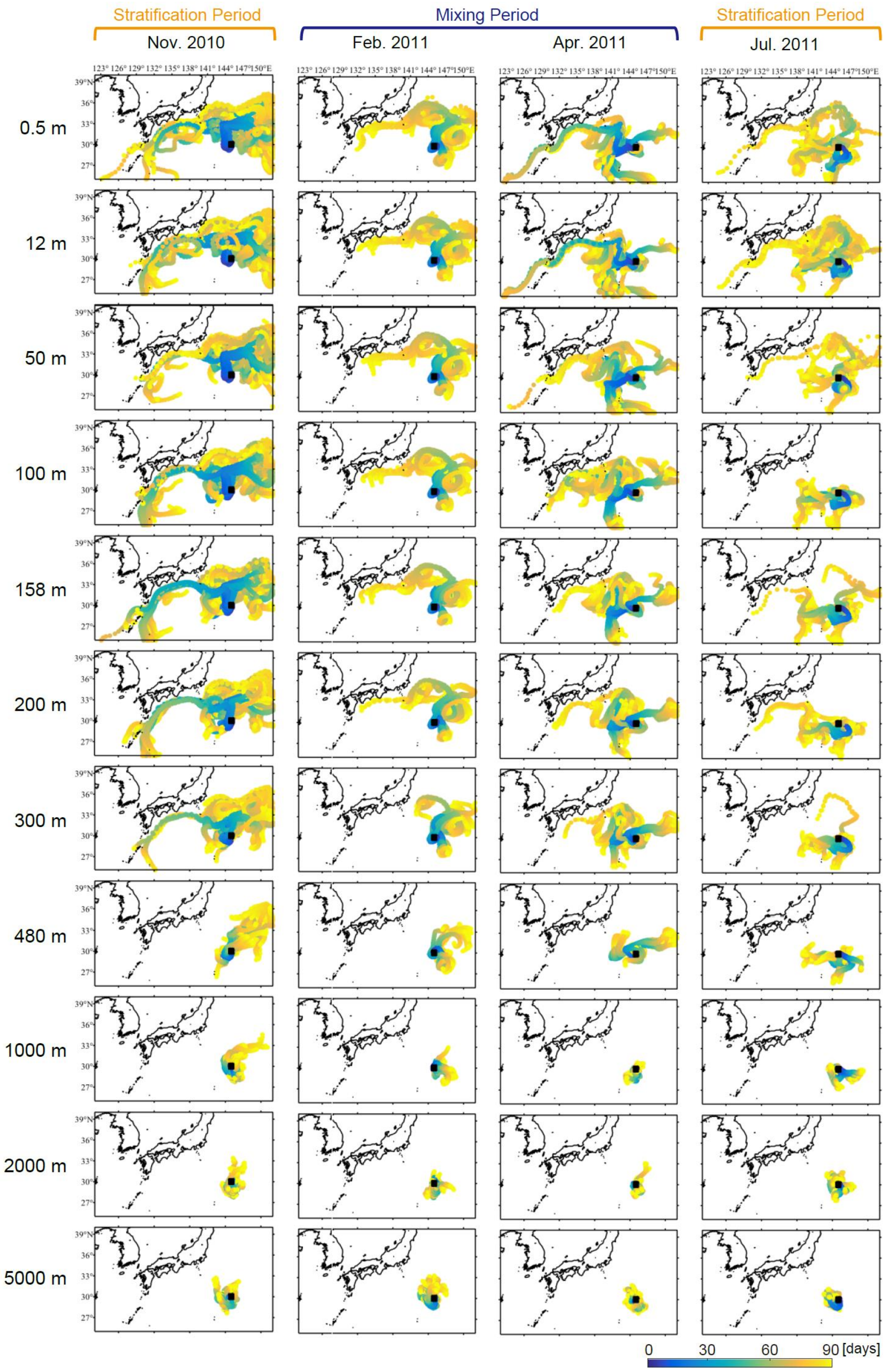

Fig S3. Back trajectories of particles present at station S1 at the various sampling depths over the 90 days prior to sampling at each month.

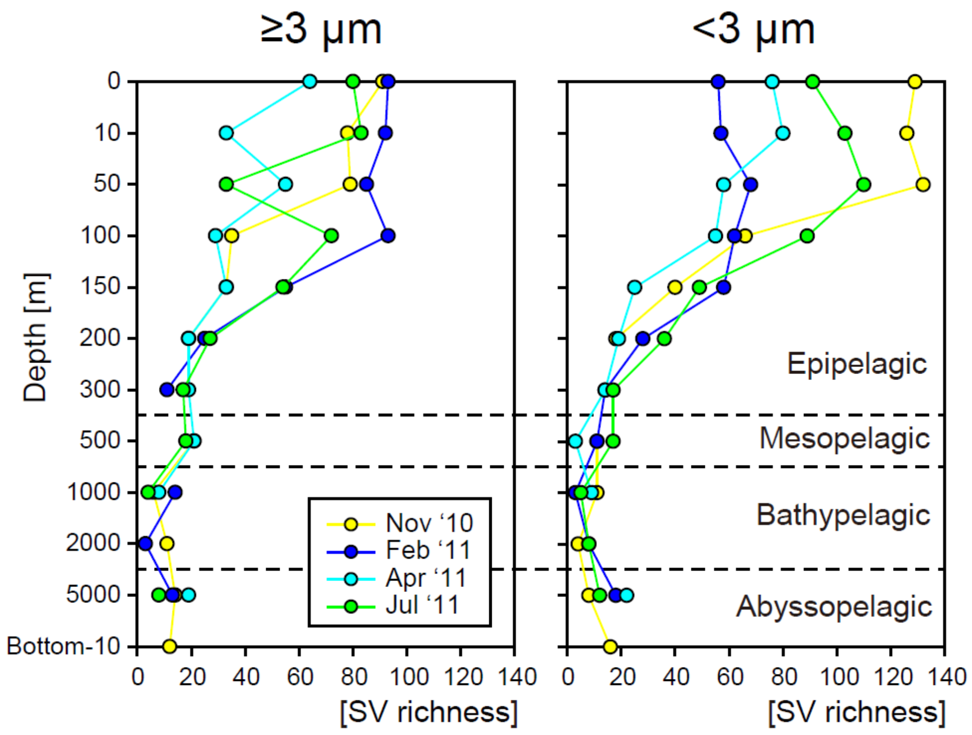

Fig S4. Vertical distributions of the SV number for each month and size fraction.

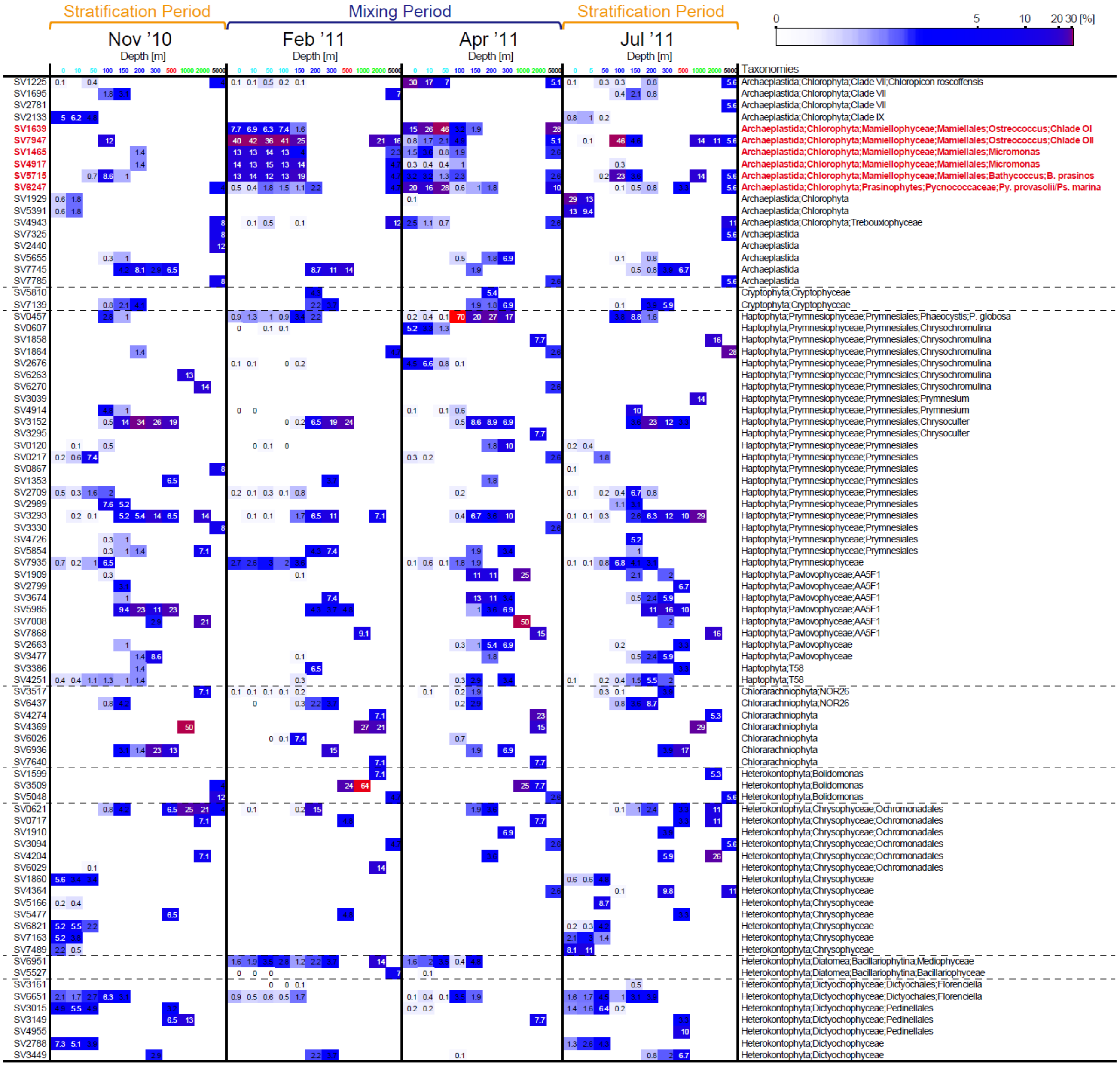

Fig S5. Heatmap of the relative abundance of representative SVs (≥ 5% of total reads at a given depth) in the < 3-µm size fraction. The relative abundance is shown in white when greater than 5%.
